# Children’s Independent Mobility to School in Seven European Countries: A Multinomial Logit Model

**DOI:** 10.3390/ijerph17239149

**Published:** 2020-12-07

**Authors:** Houshmand Masoumi, Martin van Rooijen, Grzegorz Sierpiński

**Affiliations:** 1 Center for Technology and Society, Technische Universität Berlin, 10623 Berlin, Germany; 2 Department of Transport and Supply Chain Management, College of Business and Economics, University of Johannesburg, Johannesburg 2006, South Africa; 3 Department of Education, University of Humanistic Studies, 3512 HD Utrecht, The Netherlands; martin.vanrooijen@phd.uvh.nl; 4 Faculty of Transport and Aviation Engineering, Silesian University of Technology, 44-100 Gliwice, Poland; grzegorz.sierpinski@polsl.pl

**Keywords:** children’s independent mobility, built environment, physical activity, school transport, sustainable mobility

## Abstract

The determinants of children’s independent school mobility and the contextual discrepancies between these determinants have not been comprehensively investigated in previous studies. It is important to examine these determinants because independent school mobility is associated with children’s physical activity, according to the literature. This paper examined the associations of different groups of variables such as household, mobility, perceptions, and the built environment with independent school mobility of children between 9 and 12 years using a sample of 1304 girls (50.9%) and boys (49.1%) in seven European countries. The sample was analyzed by Multinomial Logistic Regression, Chi-square test of independence, and Proportional Reduction in Error methods. According to the findings, father’s and mother’s commute mode choice, child’s mode choice of commute to school, child’s bike ownership, parent’s perception of safety, parent’s evaluation of bike lane and sidewalk quality, child’s commute distance, number of driving licenses in the household, accessibility of public transport, and population density in the neighborhood and around the school proved to be very strong and significant determinants of children’s independent school mobility in the Europe-wide sample. The comparison of the levels of independent school mobility did not show any significant differences between high-income countries such as Germany, Italy, and the Netherlands, and emerging economies and developing countries like Poland, Greece, Turkey, and Croatia. However, a direct comparison between Poland (emerging economy) (33.6%) and the Netherlands (high-income) (31.7%) revealed significant differences in the level of independent school mobility. This study found the motives for this discrepancy due to the significant difference in bike ownership, the number of household members working outside of the house, household size, commute distances of parents, and driving license possession.

## 1. Introduction

The term children’s independent mobility (CIM) is used to describe independent travel to school without adult supervision. Due to its regularity, commuting to school may have a major impact on the development of transport systems in cities. On the one hand, trips by individual cars during the morning peak hour increase the risk of congestion in the immediate vicinity of a school [1] and thus reduce traffic safety. On the other hand, independent travel, such as walking and cycling, require spatial arrangements, while bus and tram transportation necessitates the development of public transport networks. Children who are free to play outdoors and use active transport modes (walking and biking) for urban travels without the supervision of adults show higher levels of physical activity compared to those children who do not [2,3,4]. From the point of view of appropriate child development, independent travel at a very young age supports better orientation in space, e.g., familiar urban space between home and school, enhances physical fitness, and promotes interaction with other children [5]. This promotes not only physical activity but also mental health. It also encourages the use of transport modes other than individual cars.

CIM research findings from various countries are inconsistent. Moreover, we know little about several contexts and cultures in different European countries. We may approach the issue from various points of view, taking into consideration a multitude of factors. Cultural differences in particular European countries makes it quite difficult to develop a consistent approach to the issue. The CIM may depend on local customs, standards of living, parental wealth, as well as parental free time, and the most common style of upbringing in a given region. For instance, the perceptions of parents about the safety and security of children and adolescents may vary in different countries and regions. Or the way families live or move, i.e., the way they go to school or go shopping may be different in different contexts. Such cultural and geographic differences can affect the independence of school travels.

Laws are yet another factor determining transport behaviors in terms of modes selected for children to commute to their schools. Various regulations may be decisive regarding the choice between independent and dependent children’s mobility. For instance, the law may require children to be brought to school by a dedicated school bus or the so-called walking school bus, i.e., a group of children walking to school along a pedestrian lane or a sidewalk with accompaniment of one or two adults. In some countries, the law requires cities and metropolises to provide free public transport to school beyond a certain distance, e.g., Finland and Poland [6].

Promotion of the CIM requires cities to implement a number of measures. Several countries decided to develop transport education which begins at the primary school level. For instance, Finland has been providing traffic safety education since 1930, France since 1950, and Great Britain since 1974. Other solutions applicable to children of 12–14 years of age include bicycle riding courses (schools organize extracurricular classes). Such a course ends with awarding a bike license after children take an examination in real traffic. Transport education helps to develop desired behaviors at a young age. These include behavior at pedestrian crossings, understanding of traffic lights, traffic observation, etc. Additionally, crossing guards help children to cross streets in the immediate vicinity of schools.

The objective of the study is to investigate factors determining independent school mobility of 9 to 12-year-old children in Europe (Italy, Germany, Greece, Croatia, The Netherlands, Poland, Turkey), as well as to examine context bias factors through a cross-contextual comparison. Identified dependencies may facilitate decisions made by local governments and policy-makers. This study is a part of a project titled “Multisport Against Physical Sedentary” (M.A.P.S.) funded by the European Commission. It was conducted between January 2016 and December 2017.

The article discusses three research questions: what are the determinants defining children’s independent travel to school in Europe while taking into consideration different cultural contexts? Are there differences between the levels of children’s independent mobility to school in different European contexts and economies? And finally, are there significant differences between levels of children’s independent school mobility in Poland and the Netherlands, as the cases selected representing emerging markets and high-income European countries? If yes, what determinants explain these differences? Answers to these questions should help to establish the relationship between children’s mobility (independent and dependent) and other factors, such as culture and economy, in European countries (e.g., high-income countries, emerging economies, and developing countries). At the same time, it should be noted that the choice of transport mode is important not only for children and their safety but also has implications on a broader scale, since a large number of parents driving their children to school may contribute to congestion in the transport network. For this reason, the issue is very important and needs to be resolved in terms of parent decisions and transportation behaviors in the context of the children’s independent mobility.

Considering the above, the study tests the assumption that the independent children’s mobility in Europe among children of nine to twelve years of age is a complex phenomenon associated with several socio-economic, demographic, built environment, and transportation-related factors. Moreover, it is hypothesized that the level of independence varies in different European economic contexts. For the purposes mentioned above, the article has been divided into several parts. The first part reviews the status regarding the role of parents in children’s mobility. Particular attention is put on two countries, the Netherlands and Poland, with a number of references made to these countries in the following parts of this article. The next part describes the research methodology applied in nine cities and seven European countries. Findings of the project helped to identify factors determining independent mobility and analyze research hypotheses. The final part of the article discusses research findings with reference to the scientific literature and limitations determined by economic differences.

## 2. Children’s Independent Mobility and the Role of Parents

Research implemented in Denmark, Finland, Great Britain, and Norway showed that the distance to school has been growing, which results in a reduced number of trips made by bicycle and walking in favor of motorized trips (public transport included) [5]. The main reasons for this situation are urban sprawl and the growing number of private schools. Interesting research was done within the SAFEWAY2SCHOOL project, in which researchers compared bus travel to school in several European countries including Austria, Italy, Poland, and Sweden. The project identified specific needs and challenges and highlighted the importance of a discussion involving parents, children, and school administrators regarding mobility education [7]. Such investigations have already been conducted on case studies in Western Europe, but contexts in Central and Eastern Europe have a smaller share. In the following segments, the findings of previous studies in the Netherlands and Poland representing high-income western countries and emerging markets in central and eastern Europe have been presented.

### 2.1. Related Literature in the Netherlands

In the Netherlands, 90 percent of primary school students live within walking distance of the school location (1 km) and 97 percent are within biking distance, which is defined as less than 2 km. About 30 percent of the children are brought to school by car, the rest are walking or biking, alone or with parents or other attendees [8]. Twelve percent of the children are always accompanied to school by their parents, 49% are accompanied on some occasions [9]. It has become clear that the distance between home and school is the most important factor in the choice of the means of transport [10]. Interestingly, from the age of 8 to 9 years, children are starting to commute independently to school, while a total of 17 percent of primary school children (4–12 years old) are going to school on their own [9]. The percentages of independent commute to school by age are, in the most recent report: 8 years 36%, 9 years 59%, 10 years 73%, 11 years 85%, and 12 years 91% [11]. Also affecting their choice of means of transport is the factor of whether parents commute to work after bringing their children to school [12]. However, in only 10 percent of all car rides to school, it is the case that parents drive to work afterward [13]. Adults, especially parents, are not aware of the abilities of children in traffic situations. Because they do not know what to expect of children, the fear of accidents affects them, leading to withholding their children’s independent mobility [10].

In thinking about if and when their children can go to school independently, parents are influenced by traffic safety the most, followed by the distance to school, and the assessment of their children’s abilities [10]. Parents have a distinct role in teaching their children to assess traffic situations by themselves. In the Handbook Design for Children [14], children from 9 to 12 years are considered capable of crossing a street by walking, although until 11 years, there is the possibility of spontaneous behavior and a longer period of reaction to threats. In this age group, biking to school requires concentration and complex situations are difficult.

Parents are affected more by their peers and others (neighbors and children) in their perception of traffic safety, than by objective data about accidents in the school neighborhood. Therefore, it has little or no effect to educate parents about the facts and statistics [15]. There is the possibility that parents contribute to a negative situation if they think the traffic situation is not favourable. They will bring their children to school, which amplifies subjective traffic unsafety, which in turn will lead to fewer experiences of children in traffic situations. This can lead to more accidents when children eventually go to school on their own. The way to break through this negative spiral is to focus on parental behavior in trying to influence them positively so they develop a realistic view of children’s school mobility [16].

### 2.2. Related Literature in Poland

In Poland, the role of parents in CIM has been regulated by the law. Provisions of the Traffic Act (Article 43 par. 1 and 3) of 20 June 1997 [17] state that a child up to the age of 7 years may use a public road only when assisted by a person of at least 10 years of age (e.g., elder sibling). The provision has a direct link with the Code of Misdemeanor Procedure (Article 89) of 20 May 1971 [18]. A person exercising care or supervision over a minor up to 7 years of age, who allows the minor to stay on a public road or a railway track alone, can be fined or reprimanded [19]. Most often, the above provision means that children should be accompanied by parents. The special role of parents and the need to promote mobility education among children is supported by statistics on pedestrian safety (including children). In 2018, Poland recorded 57 fatalities among children of 0–14 years of age, and 2958 injuries (Road Accidents in Poland in 2018 and 2019 [20]). While analyzing data from previous years, it should be noted that the number of fatalities involving children of 0–6 years has been declining (30 and 16 deaths, respectively in 2014 and 2018). However, statistics regarding road fatalities involving children 7–14 years of age show a growing trend. Last year, for instance, the number increased by four. Additionally, there is a growing number of injuries in 7–14-year-olds (over 2090 children injured). This shows the need for traffic safety improvements.

In Poland, CIM has been regulated by the Education Act of 14 December 2016 [6]. The Act defines children’s access to free school transport. According to provisions of the Act, grade I–IV primary school, grade V–VI primary school, and junior secondary school children should be able to walk to/from school within a distance up to 3 km and 4 km respectively. If the distance exceeds the above limit, the local government should provide free transportation and supervision or the reimbursement of public transportation costs in case parents use their own cars for the purpose [21]. While considering the distribution of schools in cities, the above provisions mean that less than 13% of children were entitled to free transportation in the 2018/2019 school year, including 4% of children living more than 5 km away from their primary schools [22].

## 3. Methodology

### 3.1. Data and Variables

To answer the research questions in this study, the primary data of a survey in nine European cities (Foggia, Italy; Berlin, Germany; Thessaloniki, Greece; Rijeka, Croatia; Utrecht, The Netherlands; Łódź, Poland, Konstantynow, Poland; Malatya, Turkey, and Doğanşehir, Turkey) were analyzed. The data collection was conducted in 2016. The data included the validated data of 1304 child/parent pairs, who filled out self-administered questionnaires with 26 questions about household socio-economics, mobility habits of the child and the parents, and perceptions about safety and security. No legal consent was obtained from the respondents, but they were informed about the aims and scope of the study by the teacher of the class. The teachers and school authorities were also given information about the project and the contents of the study. The study procedure was not submitted to an ethics committee, but it was attempted to not violate the privacy of the respondents, i.e., no respondent was asked for names or home addresses and when collecting the data of home addresses, only the nearest street intersection to the house was asked for.

No standardized, validated questionnaire was used, instead, a questionnaire was developed based on the existing literature on the topic. The development of questionnaires based on the necessities and objectives of the research is common in mobility, transportation, and urban research. Moreover, for fulfilling an important step in questionnaire validation, one of the main steps of validation, namely data cleaning, was cared for. For increasing the quality of the output data, two rounds of data cleaning were conducted, once by the local surveyors in each of the case cities and once by the central survey office in Berlin. Thus, less trustable data were eliminated from the dataset. The results of the literature review have already been published in peer-reviewed journal papers. These include a review of children’s active travels to school and the relations with their body weight [23] and the built environment and children’s physical activity [24].

The schools were selected from different urban forms including different accessibility to green/open space and public transport as well as different connectivity of street networks. The sub-samples of each school were selected from the classes with students of the age range of the study (9–12 years). The survey led to an overall response rate of 52 percent (Italy: 89.06%, Germany: 32.17%, Greece: 89%, Croatia: 92.59%, the Netherlands: 26%, Poland: 28.33%, and Turkey: 100%). The data collection based on the questionnaire was done by asking the parents of children to fill out a form. Considering the age of the pupils (9–12 years), they were not asked directly. Instead, their parents filled out the questionnaires on their behalf. Only two of the questions about children’s perceptions of safety and security were asked directly to the pupils, while all the other questions were answered by the parents. The parents of children in each class were asked by the teachers to fill out the questionnaire. Traditional paper-and-pen questionnaires were used to collect self-reported data from the parents of the pupils.

The urban form traits in the vicinity of the schools were collected by the project collaborators located in different countries (Italy, Germany, Greece, Croatia, the Netherlands, Poland, and Turkey) as well as Google Maps. These characteristics consisted of aggregate data about the distance from the nearest intersection to homes to the nearest intersection to school (commute distance), the number of street crossings, street connectivity, accessibility to public transportation (PT), population density, and the number of public open/green spaces. These data were gathered for catchment areas of 3 by 3-km rectangles around the schools. The data was collected from students of 21 schools throughout Europe. Full details of the questionnaire, the survey methods, and the results including representativeness, city-wide response rates, and survey loss have already been published as an open-access research paper [25].

The output data of the survey contained different types including dichotomous, categorical, and continuous. For the sake of consistency, in modeling and analyzing as well as for making the outputs of logistic modeling more presentable, the continuous data were changed into categorical or dummy data. Table 1 presents the variables that were identified as appropriate for analysis because of a preliminary understanding of them and/or the emphasis of the existing literature about their association with children’s independent school commuting. Some of these data were originally categorical or binary in the questionnaire. Those that were continuous were transformed into categorical variables. The transformed variables were the number of people working outside of the house, household size, number of children in the household, household income, commute distance, driving license, number of street crossings, street connectivity, accessibility of public transportation (PT), population density, and public open/green spaces. The rest were already ordinal or categorical.

### 3.2. Analysis Methods

Multinomial Logistic Regression (MNL) modeling was applied to answer the first question about the effective determinants of independent school commuting. For analyzing the outputs, *p*-values of less than 0.05 were considered as significant and those between 0.05 and 0.1 were regarded as marginally significant. The dependent variable was “individuals accompanying a child to school” that included the categories of “father”, “mother”, “no one”, and “siblings/close relatives/others”. A similar model with binary categories of “dependent” and “independent” will have similar outputs, thus this configuration was chosen so that more detailed information could be generated. The model fit information shown in Table 2, shows a good fit with a *p*-value of less than 0.001 and Nagelkerke’s R^2^ of 0.69. This Pseudo-R^2^ value showed that 69 percent of the variation is explained by the model, which is considered a strong fit. With a *p*-value of 0.341, the results of the Goodness of Fit reject the hypothesis of no fit, so the model provided a good prediction of the variables.

In order to find differences in independent school commuting in different European contexts based on national economies (research question 2), the seven countries in which the survey was conducted were divided into two categories of high-income countries including Germany, Italy, and the Netherlands, and emerging economies and developing countries including Poland, Greece, Turkey, and Croatia. If a country was found in at least one of the main lists of emerging economies (International Monetary Fund, FTSE Group, Standard and Poor’s Financial Services LLC, Emerging Markets Bond Indexing Monitor, Dow Jones, Russell Investments, and Columbia Center for Sustainable Investment of Columbia University, as well as BRICKS and Next Eleven lists), then it was included in the second group. Poland, Greece, and Turkey were listed in at least one of the lists, but Croatia was only found in lists of developing countries. Table 3 shows the frequencies and percentages of responses in these two groups. Comparison between the dependence of school commuting in the two groups of countries was undertaken by use of a Chi-square test of Goodness of Fit. The null hypothesis was that there is no significant difference in the levels of independent commuting in the two groups of countries. *p*-values of less than 0.05 rejected the null hypothesis, confirming the alternative hypothesis that there is a significant difference between the observed and the expected values. This hypothesis testing was controlled by the value of Cramer’s V, which is a measure of proportional reduction in error (PRE) methods. Cramer’s V provides trusted results when there are different nominal groups on the rows and columns in the crosstab. The independence level variable had three categories: dependent, independent, and missing, so Cramer’s V test was applied and 0.05 was taken as the *p*-value significance level. Cramer’s V varies between −1 and +1, where 0 represents no association (difference) and a value of 1 shows a complete association. All the above analyses were done by IBM SPSS version 25 (Developed by IBM, Armonk, NY, USA).

To answer the third research question about the differences between the levels of independent mobility to school in Poland and the Netherlands as cases of emerging markets and high-income European countries, a Chi-square test of independence was conducted between the frequencies of independent mobility to school in the Polish and Dutch sub-samples (Utrecht in the Netherlands and Konstantynów Łódzki and Łódź in Poland). The same analysis was done using Cramer’s parameter of the Proportional Reduction in Error (PRE), where higher values of Cramer’s V measure show stronger associations. The null hypothesis was that the independent school mobility in the two sub-samples was independent. To understand the motives of the possible significant difference, the Chi-square test of independence was run for the significant variables of the Europe-wide MNL model for the two sub-samples.

### 3.3. Independent Mobility to School in High-Income Versus Emerging Market Cases

The cities of Utrecht in the Netherlands and Konstantynów Łódzki and Łódź in Poland were taken as representative cases, the independent school mobility of which was explained using the findings of the statistical analyses. The city of Utrecht is the fourth biggest city in the Netherlands and had 343,134 inhabitants at the beginning of 2017 [26]. The number of children who were going to primary school (4 to 12 years) in 2015 was 29,497 [27]. In comparison to the 50 largest municipalities in the Netherlands, Utrecht is ranked third place on the social-economic index [28]. Seventy-seven percent of the children in the two highest grades of the primary school, aged 10–12 years, were going to school on foot or by bike every day. Five percent were never commuting to school on foot or by bike. The ethnic background was a factor, with students of a Turkish background going less often on foot or by bike each day, namely, 61 percent. Children who were going to school in their own district were going more often every day to school by mode of walking or biking (80%) than children going to school from another district (56%) [29].

The town of Konstantynów Łódzki, Poland, has a population of about 18,000 [22,30] including around 3000 people below 18 years of age [31]. In the 2017/2018 school year, there were 1111 primary school children. The town is situated about 9 km from the center of Lodz, the third largest Polish city regarding its population after Warsaw and Krakow [32]. The population is 685,300, which accounts for 27.8% of the total population of the province [32]. In the 2018/19 school year, the number of primary school children was 40,840. By the end of 2018, Łodź had nearly 505,000 vehicles registered, including 399,417 passenger cars. In 2014, public transport accounted for 40% of all trips within the city, whereas walking amounted to 29%, and passenger car was 30% of the total daily trips. Excluding walking, in 2014, public transport accounted for 55% of all trips [33]. The town has been implementing a sustainable public transport plan designed to encourage a modal shift towards more eco-friendly modes to reach the 25% target for public transport by 2025. Additionally, Łódź has been operating a bike-sharing system, known as the Łódź Public Bike. In 2018, the number of rentals reached 1.6 million [32]. Considering different age groups, people of 6–24 years old preferred walking trips (53%). As many as 39% of them chose public transport, whereas passenger car trips accounted for only 15% and biking trips 6% of the total (sum total for particular modes does not add up to 100%, since people surveyed could choose more than one mode of transport). Since the youngest group members (6–15-year-olds) cannot drive a car, it might be a reason why they chose public transport and walking trips so frequently.

## 4. Findings

### 4.1. The Determinants of Independent School Transport in Europe

The MNL model provided several significant variables (Table 4). Only a few of the variables were not significant, including gender, a child’s perception of safety, the number of people working outside the home, household size, and the number of street crossings. Others were either significant at 0.01 and 0.05 levels or marginally significant (0.05 < *p* < 0.1). Father’s and mother’s commute mode choice, child’s mode choice of commute to school, child’s bike ownership, parent’s perception of safety, parent’s evaluation of bike lane and sidewalk quality, child’s commute distance, number of driving licenses in the household, accessibility of public transport, and population density in the neighborhood and around the school were very strongly significant (*p* < 0.001).

This table presents the big picture only, providing no variable coefficient values. The values of coefficients and their signs for the categories are depicted in Table 5, where only significant categories have been kept in the model. The no-response categories as well as the insignificant ones were eliminated from the model to make it more representable. According to this table, age was a significant variable: 9-year old children were 162% more likely to go to school with their fathers than 12-year olds, relative to independent mobility children. The same was true for 10-year olds traveling with their father, with a likelihood of 125%. The figures were 206%, 150%, and 90% for 9, 10, and 11-year olds respectively going to school with their mother. Those who commuted with their father by bike, on foot, or by family car were 1.43, 1.25, and 1.05 times more than those who commuted by public transport with their fathers respectively compared to those who go independently. The association between the mothers’ choice of commuting mode and them being unemployed was also significant. Children whose mothers commuted by foot were 1.05 times less likely to go to school with their mother compared to those whose mothers stayed at home. The findings regarding the children’s choice for commuting were: those who commuted with their fathers were more than five times less likely to do it on foot, by bike, by private/school service, or by public transport. The figure was more than three times higher for those going with their mothers. Likewise, it was two or more times more likely that children were accompanied to school by siblings, relatives, and others when the child’s commuting mode was by bike, walking, or public transport, compared to being taken by car. These findings indicated that if children commuted with their parents, it was highly probable that they were chauffeured to school. This showed the importance of their dependence on parents’ commuting habits. The same could be observed about parents’ perceptions. When the parents perceive the environment (neighborhood, the route to school, etc.) as unsafe for the child (regarding threats from other people), fathers were more than two times more likely to take their child to school compared to parents who felt the environment was safe. Children whose parents believed the environment was safe or very safe (regarding traffic accidents) were more than 1.3 times less likely to commute with their mother compared to those who thought the environment was unsafe. This association was stronger for fathers. If parents thought the area was moderately safe or unsafe, then it was respectively 1.27 and 2 times less likely that they would trust siblings or others to take the child to school. Additionally, the structure of families mattered; children of families in which nobody worked were 7% less likely to commute with their mothers compared to children of families in which one person worked. As expected, children of households with four members or more were 44% less likely to commute with their mother compared to those with two or three children. Families with one child were 24% less likely to let their child go to school with relatives or others compared to those who had two or three children. When the number of driving licenses in a household increased, children were less likely to commute with their mothers, because there were other members who could take them to school by car. Household income was only significant regarding siblings/relatives/others; families with an average monthly income of more than 4000 € were 67% less likely to send their children to school accompanied by siblings, relatives, or others compared to those with an income of between 500 € to 4000 €.

The findings regarding land use and urban form were also noteworthy. When there were three or less street-crossings between the home and the school, children were 43% to 46% less likely to commute with their fathers or mothers compared to when there were more than four crossings. This reflected the concerns of parents regarding the safety of crossing the streets alone. Siblings and others accompanied children 23% less in areas with three or less crossings compared to areas with four to nine crossings. When accessibility of public transportation was low, children were 1.6 times less likely to commute with their father compared to those with medium accessibility. When population density was low, they commuted with their fathers 13% more compared to those in medium density. If the population density was high, they were less likely to commute with their mother compared to medium densities. Children would be taken to school by siblings and others about two times less than when the population density was high compared to when it was medium density. In neighboring areas of schools with fewer open or green spaces, children were 21% less likely to be accompanied by their fathers and 49% less likely to be taken by their mothers compared to areas with a medium number of public spaces.

### 4.2. Contextuality of Independent Mobility to School

The above model depicts the associations of different factors with dependent or independent transport to school in the European context. The model provides a big picture of the topic within a very wide range of cultures, geographies, and climates from Utrecht, the Netherlands to Malatya, Turkey, and from Łódź, Poland to Foggia, Italy. As stated at the beginning of this paper, the socio−cultural motives of differences in the levels of dependent school mobility have not been thoroughly examined in different cultures. Here, we only focus on the economic status of the European countries as a determinant of the societal differences between contexts.

As explained in the methodology section, the Chi−square test was applied to test the significant associations between the two groups of countries including high−income countries and emerging/developing countries in Europe. The *p*−value of the Chi−square Goodness of Fit test was less than 0.05, so the null hypothesis of no association was rejected. In other words, the levels of dependent (or independent) school transport in the two groups of countries are similar. This finding did not show the levels of association. To understand the association level, the PRE-test of Cramer’s V was applied to the two groups of economies to investigate the similarities between the independence levels. The result showed that the association was only 15.8%, which was considered to be a weak similarity. This finding is of remarkably high statistical significance (*p* < 0.001). It was still necessary to check the frequencies to understand the patterns and distributions better. More than 52% of children in developing countries and emerging markets had dependent mobility to school, while this figure was 46% in high-income countries. In other words, the independent school mobility of children was higher in wealthier European countries. This difference was statistically insignificant.

In order to understand the possible difference between independence of school mobility in Poland, as a representative of European emerging markets, and the Netherlands as a high-income country (research question 3), the Chi-square test of independence and Cramer’s V tests were run for the two countries. The Pearson Chi-square measure did not show any association between the dependent school mobility in the respondents of the two countries (*p* = 0.927) and the Cramer’s V value was not significant. In other words, independent school mobility in Poland (33.6%) was slightly more than in the Netherlands (31.7%). This made the overall difference between the dependency of school mobility significantly different in the respondents of the two countries.

The second part of question 3 of the study explored the differences between the possible variables that caused the significant difference in children’s dependence. Exploring the above differences in the dependent mobility in the Polish and Dutch sub-samples assisted in finding some of the differences in the significant variables of the Europe-wide MNL model. Significant differences could be found: in children’s bike ownership (χ^2^ = 4.42, *p* = 0.109), where Dutch children had more bikes; in the number of household members working outside of the house (χ^2^ = 6.27, *p* = 0.099), where Dutch family members worked more (two or more family members of 55% of the Dutch respondents worked outside the house compared to 48% in Poland); in household size (χ^2^ = 5.39, *p* = 0.067), where the Dutch households were larger; in commute distances of parents (χ^2^ = 1.76, *p* = 0.624), where Dutch families commuted slightly significantly longer distances, and; in driving license possession (χ^2^ = 0.571, *p* = 0.751), where Dutch families had more driving licenses. As cultural and lifestyle issues, the above variables were considered to be the factors that motivated the significant differences in independent mobility to school in Poland and the Netherlands. These data answered the second part of the third research question of this study.

## 5. Discussion

This study indicates that safety is a major concern behind the decision made by parents regarding their children’s mobility. The findings of this study show that the father’s and mother’s commute mode choice, the child’s choice of commute mode to school, the child’s bike ownership, parent’s perception of safety, parent’s evaluation of the sidewalk quality, the child’s commute distance, the number of driving licenses in the household, accessibility to public transport, and population density in the neighborhood and around the school are very strong and significant determinants of children’s independent school mobility in the Europe-wide sample. The comparison of the levels of independent school mobility did not show any significant differences between high-income countries such as Germany, Italy, and the Netherlands, and emerging economies and developing countries like Poland, Greece, Turkey, and Croatia. However, a direct comparison between Poland and the Netherlands revealed significant differences in the level of independent school mobility. These results regarding the determinants as well as the differences between disparities of independent school mobility in emerging markets and high-income European countries give us insight into the necessities of local urban and transport planning for the promotion of CIM.

Interestingly, there is not much research on independent mobility in the Netherlands. Most research is undertaken on a local level, and there is no actual data available. Although there is a good overview of childrens commute mode choice in Utrecht—walking or biking—there is no data available on whether or not they are going alone. Further research on this topic could be valuable on gaining insight into how freely children are going to school and which factors are influencing this.

The necessity of having children learn to assess traffic situations and enhance their abilities in going to school on their own is often stated in Dutch policy documents. However, the programs seem to be fragmented as many stakeholders are involved and action is taken mainly on a local level and not coordinated nationwide. Stakeholders include primary schools, the municipality, police, parents, and organizations in traffic safety, cycling promotion, and health initiatives. On the policy level, the topic of children’s independent mobility is divided into diverse branches: public health, active youth, education, transport and traffic, public spaces, district design, and new housing. This makes it more difficult for municipalities to develop an integral approach. Nevertheless, key stakeholders are the children themselves [34]. By involving them and discussing the subject with them, children can be the best ambassador for their own need for increased freedom and independence. They can influence their parents from their perspective and have the tools to persuade them to let them go to school on their own.

The network of child-friendly cities is a key stakeholder to influence policy and make suggestions for research on this subject. In the Netherlands, municipalities can join this network and exchange experiences and good practices from each other and in international contexts [35]. A proactive role of a municipality, not only regarding traffic safety but also towards a healthy active lifestyle and sustainability, can support schools and parents in promoting the independent mobility of children on their way to and from school [36].

In Poland, attention has focused recently on traffic safety, especially the safety of children. However, there is a shortage of wider research designed to identify children’s mobility behaviors and their underlying factors. In this context, the attempt to find answers to the research questions can be treated as a pilot study. As mentioned earlier, over 87% of school-age children live 3 km away from their schools. Leaving health issues aside, this translates into an opportunity to promote desired children’s mobility. Thus, local governments should pay special attention to the development and promotion of safe bike routes. According to the survey, this particular commuting mode is used in Konstantynów Łódzki by only 5% of children compared to 12% in Łódź. This is much lower, for instance, than in Utrecht (34%) [23].

At the moment, in both Polish cities included in this study, the majority of children are driven to school by their parents (respectively 24.8% and 33.3%). Such commuting behavior has become one of many factors contributing to traffic congestion. In certain Polish cities (e.g., Kraków, Poznań, and Łódź), the issue has been recognized and, following the example of Vienna, these cities are considering introducing a ban on passenger car traffic in zones around schools during the morning peak [37].

The results described in the findings section show a strong relationship between children’s mobility and the perception of safety near schools by their parents. This might be yet another argument for local governments to introduce measures that promote independent children’s mobility. Cities have been implementing solutions to calm traffic in the vicinity of schools. The main goal is to enhance safety by limiting speed and shifting transit traffic to other sections of the transport network [38].

In Finland, the survey shows a strong relationship between children’s mobility and the perception of traffic safety among parents [39]. A factor limiting the choice of active travel modes (walking or cycling) is the density of built-up development. At the same time, no major differences are observed regarding child age and gender. The research described in the article produces similar results regarding gender. However, a decision regarding independent mobility is age bias. For instance, in Norway, it has been observed that boys are engaged in independent mobility more frequently than girls [9].

Concerning CIM, the distance between home and school is yet another relevant factor in other parts of Europe and other continents. In Hong Kong (a high-income country), almost one-third of children are mobile independently [40]. This corresponds with the findings of this study, where 33 and 31 percent of children go to school independently in Poland and the Netherlands, respectively. Several of the significant determinants of CIM in the European sample of this study are also significant in Hong Kong, e.g., distance to school, age, neighborhood settlement types (in our sample: street connectivity, accessibility of public transportation, and availability of open spaces), and density are significant in both Europe and Hong Kong, but household income is significant in Hong Kong but only marginally significant in Europe. Moreover, several other variables that were found significant in this study were not found important in Hong Kong [40]. In line with the findings of our study, the perceptions of parents concerning security in the neighborhood are among the determinants of children’s autonomous mobility to school [40]. In Finland, although nearly every child makes independent trips to school by walking and cycling [40], the number of such trips is inversely proportional to the distance. A US study in California shows a significant relationship between independent mobility and distance. If the distance is about half a mile, 75% of children make independent trips to school, whereas in the case of 1–1.5 miles, it is only 18% [41].

In terms of correlations of the neighborhood-level land use and urban form with independent school mobility, the general findings of this study on European cities confirm the results of a previous study in Taipai that found a relation between neighborhood environment e.g., sidewalks, smaller residential blocks, low density of street intersections with independent school travel [42]. Likewise, the study found a correlation between the independence level with street connectivity, accessibility of PT, population density, and availability of open spaces. According to the findings of this study, accessibility to PT is correlated with independent school travel. This finding is in line with the conclusion of Mackett, who found decreasing diversity of travel modes causes parents to take their children to school by private modes [41]. In our study in Europe, the number of children in the household is correlated with independent school mobility (but not the household size itself). This finding is in general accordance with the result of Parish and Cloud, who noted that children from single-parent families are more likely to have independent school travel than children living with two parents [43]. In the overall sample of this study in seven European cities, mothers take their children to school more than fathers (26.2% vs. 12.2%), just as Vovsha and Peterson showed a higher probability of mothers chauffeuring their children to school [44].

To sum up, independent children’s mobility and its underlying motives, such as parent perception, etc. are relatively context-specific. In other words, the context-related cultural issues are decisive regarding safety perception and its outcomes, such as active travel to school and, consequently, improved physical fitness among children. This study does not find significant differences between independent school mobility of children in developing countries/emerging markets and high-income countries as a whole, but some significant differences were found between cases from the two groups of economies, exemplified by Poland and the Netherlands. This indicates the possibility of finding several other significantly different comparisons. Thus, in order to increase the physical activity of children, the local barriers and motives of independent mobility to school should be studied at the local scale.

Finally, the significant discrepancies of the five variables of bike ownership, the number of household members working outside of the house, household size, commute distances of parents, and driving license possession in the Polish and Dutch sub-samples necessitate a causal relationship between these variables and CIM. This relation is justified by the methodology of this study. These five variables were firstly identified as significant determinants of independent school mobility in Europe and were subsequently shown to have significantly different values in Poland and the Netherlands. Thus, it is logical to conclude that they caused the significant differences in CIM.

The limitation of this study was the lack of resources for the production of the built environment disaggregate data. In future studies, with the presence of the necessary base maps, time, and human resources, it will be preferable to generate land-use variables based on disaggregated data. This will result in a more robust and higher power of output models and tests.

## 6. Conclusions

This study identified several variables as significant determinants of independent school mobility: father’s and mother’s commute mode choice, child’s commute mode choice to school, child’s bike ownership, parent’s perception of safety, parent’s evaluation of bike sidewalk quality, child’s commute distance, number of driving licenses in the household, accessibility to public transport, and population density in the neighborhood and around the schools. These associations have been identified as important in a sample distributed from the Netherlands to Turkey, so it is probable that there is a difference between contexts. According to the hypothesis testing of this study, there is no significant difference between the two groups of countries based on their economy (developing/emerging markets and high-income countries). Nevertheless, the two countries selected as example cases showed a significant difference in the levels of independent mobility (Poland higher than the Netherlands). In the search for possible reasons, significant differences were found between the frequencies of responses in the Polish and Dutch sub-samples regarding the determinants of independent school mobility of children. These variables included bike ownership, the number of household members working outside of the house, household size, commute distances of parents, and driving license possession. As seen in the discussion section, the difference in CIM of Poland and the Netherlands has been “caused” by these five variables, so they can be applied in the implementation of school mobility programs and projects to increase autonomous school mobility.

## Figures and Tables

**Table 1:** Categorical variables applied in this study and their frequencies and marginal percentages (*n* = 1304).

Variable	Category	*n*	%	Variable	Category	*n*	%
Individuals accompanying child	no response	88	7.1%	Parents’ perception of security	no response	50	4.0%
	father	151	12.2%		insecure	171	13.8%
	mother	324	26.2%		moderate	458	37.1%
	no one	519	42.0%		secure	386	31.3%
	siblings/close relatives/others	153	12.4%		very insecure	71	5.7%
Age	9	137	11.1%		very secure	99	8.0%
	10	445	36.0%	Parents’ self-evaluation of sidewalk quality	no response	52	4.2%
	11	426	34.5%		dissatisfied	279	22.6%
	12	227	18.4%		indifferent	263	21.3%
Gender	no response	1	0.1%		satisfied	443	35.9%
	female	628	50.9%		very dissatisfied	92	7.4%
	male	606	49.1%		very satisfied	106	8.6%
Father’s commute mode choice	no response	86	7.0%	Parents’ self-evaluation of bike path quality	no response	60	4.9%
	bike	67	5.4%		dissatisfied	392	31.7%
	by foot	94	7.6%		indifferent	228	18.5%
	car	632	51.2%		satisfied	219	17.7%
	he doesn’t work	77	6.2%		very dissatisfied	293	23.7%
	public transport	279	22.6%		very satisfied	43	3.5%
Mother’s commute mode choice	no response	93	7.5%	No. of people working outside of the house	no response	35	2.8%
	bike	73	5.9%		≥2	604	48.9%
	by foot	131	10.6%		0	77	6.2%
	car	294	23.8%		1	519	42.0%
	public transport	248	20.1%	Household Size	no response	10	0.8%
	she doesn’t work	396	32.1%		≥4	934	75.6%
Shopping in the neighborhood	no response	45	3.6%		1	3	0.2%
	50–50	165	13.4%		2–3	288	23.3%
	always	272	22.0%	No. children in the household	no response	7	0.6%
	never	39	3.2%		≥4	105	8.5%
	sometimes	268	21.7%		0	3	0.2%
	usually	446	36.1%		1	289	23.4%
Entertainment in the neighborhood	no response	41	3.3%		2–3	831	67.3%
	50–50	177	14.3%	Income	no response	280	22.7%
	always	92	7.4%		≤500 €	32	2.6%
	never	147	11.9%		>4001 €	118	9.6%
	sometimes	389	31.5%		501–4000	805	65.2%
	usually	389	31.5%	Commute distance	no response	269	21.8%
Child’s travel to school mode	no response	52	4.2%		801–2500 m	166	13.4%
	bike	87	7.0%		Less than 800 m	793	64.2%
	by foot	718	58.1%		>2501 m	7	0.6%
	by private/school service	50	4.0%	Driving license	0	60	4.9%
	by PT	96	7.8%		1	452	36.6%
	own car	232	18.8%		2 or more	723	58.5%
Child’s bicycle ownership	no response	10	0.8%	No. of street crossings	no response	59	4.8%
	no	249	20.2%		≤3	648	52.5%
	yes	976	79.0%		≥10	120	9.7%
Child’s perception of safety	no response	46	3.7%		4–9	408	33.0%
	moderate	422	34.2%	Street connectivity	High	358	29.0%
	safe	377	30.5%		Low	475	38.5%
	unsafe	164	13.3%		Medium	402	32.6%
	very safe	134	10.9%	Accessibility to PT	High	315	25.5%
	very unsafe	92	7.4%		Low	410	33.2%
Child’s perception of security	no response	50	4.0%		Medium	510	41.3%
	insecure	156	12.6%	Population density	High	280	22.7%
	moderate	404	32.7%		Low	451	36.5%
	secure	418	33.8%		Medium	504	40.8%
	very insecure	85	6.9%	Open spaces	High	320	25.9%
	very secure	122	9.9%		Low	491	39.8%
Parents’ perception of safety	no response	47	3.8%		Medium	424	34.3%
	moderate	447	36.2%	Valid		1235	100.0%
	safe	341	27.6%	Missing		69	
	unsafe	206	16.7%	Total		1304	
	very safe	99	8.0%	Subpopulation		1232	
	very unsafe	95	7.7%				

**Table 2:** Model fitting information and goodness of fit of the Multinomial Logistic Regression (MNL) model.

Measure	Model	Model Fitting Criteria	Likelihood Ratio Tests		
		−2 Log Likelihood	Χ^2^	df	*p*-Value
Model Fitting Information	Final	2,208,249	1,294,537	364	<0.001
Goodness of Fit	Measure	Chi-Square	df	*p*-value	
	Pearson	4598.669	4560	0.341	
	Deviance	2205.477	4560	1	

Pseudo R^2^: Nagelkerke = 0.69.

**Table 3:** Dependent and independent school commuting in high-income countries (Germany, Italy, and the Netherlands) versus emerging/developing countries (Poland, Greece, Turkey, and Croatia) in the sample (*n* = 1304).

Measure			Individuals Accompanying Child to School					Total
			No Response	Father	Mother	No One	Siblings/Close Relatives/Others	
Economy Status	Developing/Emerging Country	*n*	34	93	197	357	139	820
		%	4.1%	11.3%	24.0%	43.5%	17.0%	100.0%
	High Income Countries	*n*	61	65	141	201	16	484
		%	12.6%	13.4%	29.1%	41.5%	3.3%	100.0%
Total		*n*	95	158	338	558	155	1304
		%	7.3%	12.1%	25.9%	42.8%	11.9%	100.0%

**Table 4:** The significance of variables of the MNL model with the dependent variable of “individuals accompanying a child to school” (*n* = 1304).

Effect	Likelihood Ratio Tests			
	Model Fitting Criteria	Likelihood Ratio Tests		
	−2 Log Likelihood of Reduced Model	Χ²	df	*p*-Value
Intercept	2208.24	0.000	0	
Age	3143.25	935.009	12	<0.001
Gender	2211.64	3.392	8	0.907
Father’s commute mode choice	14,111.69	11,903.443	20	<0.001
Mother’s commute mode choice	2292.63	84.380	20	<0.001
Shopping in the neighborhood	2236.98	28.735	20	0.093
Entertainment in the neighborhood	2246.03	37.782	20	0.009
Child’s travel to school mode	2494.76	286.508	20	<0.001
Child’s bicycle ownership	4143.86	1935.615	8	<0.001
Child’s perception of safety	2227.58	19.335	20	0.500
Child’s perception of security	2238.97	30.725	20	0.059
Parents’ perception of safety	2634.29	426.044	20	<0.001
Parents’ perception of security	2245.29	37.042	20	0.012
Parents’ self-evaluation of sidewalk quality	6591.01	4382.768	20	<0.001
Parents’ self-evaluation of bike path quality	2244.74	36.493	20	0.013
No. of people working outside of the house	2219.67	11.422	12	0.493
Household Size	2222.5	14.252	12	0.285
No. Children in the household	2238.5	30.251	16	0.017
Income Groups	2227.75	19.504	12	0.077
Commute distance (distance to school)	8425.98	6217.730	12	<0.001
Driving license	2236.24	27.998	8	<0.001
No. of street crossing	2226.62	18.380	12	0.105
Street connectivity	2225.14	16.893	8	0.031
Accessibility to PT	2245.45	37.203	8	<0.001
Population density	2238.59	30.341	8	<0.001
Open spaces	2229.07	20.818	8	0.008

**Table 5:** MNL model explaining the coefficients and significance of response categories with the reference-dependent category of independent mobility to school (no one accompanying) (*n* = 1304).

Family Members Accompanying Child	Category	B	Wald	*p*-Value	Exp(B)	Family Members Accompanying Child	Category	B	Wald	*p*-Value	Exp(B)
father	Intercept	−10.905	38.24	<0.001			Child’s perception of security = very insecure	−1.965	7.136	0.008	0.14
	Age = 9	1.619	10.32	0.001	5.046		Child’s perception of security = very secure	Reference Category			
	Age = 10	1.249	9.511	0.002	3.488		Parents’ perception of safety = moderate	−1.088	4.065	0.044	0.337
	Age = 12	Reference Category					Parents’ perception of safety = very unsafe	Reference Category			
	Father’s commute mode choice = bike	1.432	4.765	0.029	4.189		Parents’ self evaluation of bike path quality = dissatisfied	1.154	3.242	0.072	3.17
	Father’s commute mode choice = by foot	1.248	5.647	0.017	3.484		Parents’ self evaluation of bike path quality = indifferent	1.466	4.928	0.026	4.334
	Father’s commute mode choice = car	1.059	7.108	0.008	2.884		Parents’ self evaluation of bike path quality = very dissatisfied	1.08	2.912	0.088	2.945
	Father’s commute mode choice = he doesn’t work	2.128	15.9	<0.001	8.395		Parents’ self-evaluation of bike path quality = very satisfied	Reference Category			
	Father’s commute mode choice = public transport	Reference Category					No. of people working outside of the house = 0	−0.929	4.805	0.028	0.395
	Child’s travel to school mode = bike	−5.104	50.49	<0.001	0.006		No. of people working outside of the house = 1	Reference Category			
	Child’s travel to school mode = by foot	−5.251	85.51	<0.001	0.005		Household Size >= 4	−0.66	3.444	0.063	0.517
	Child’s travel to school mode = by private/school service	−5.016	40.07	<0.001	0.007		Household Size = 2−3	Reference Category			
	Child’s travel to school mode = by public transport	−6.579	74.83	<0.001	0.001		Driving license = 0	1.261	8.096	0.004	3.53
	Child’s travel to school mode = own car	Reference Category					Driving license = 1	0.446	3.945	0.047	1.561
	Parents’ perception of safety = moderate	−1.701	7.459	0.006	0.182		Driving license = 2 or more	Reference Category			
	Parents’ perception of safety = unsafe	−1.698	7.579	0.006	0.183		No. of street crossing < =3	−0.643	8.08	0.004	0.526
	Parents’ perception of safety = very unsafe	Reference Category					No. of street crossing = Between 4 and 9	Reference Category			
	Parents’ perception of security = insecure	2.142	4.183	0.041	8.514		Accessibility to PT = Low	−0.81	5.785	0.016	0.445
	Parents’ perception of security = very secure	Reference Category					Accessibility to PT = Medium	Reference Category			
	Commute distance = 801−2500 m	15.261	1261.9	<0.001	4245000		Population density = High	−0.61	3.874	0.049	0.544
	Commute distance = more than 2501 m	Reference Category					Population density = Medium	Reference Category			
	No. of street crossing < =3	−0.67	4.879	0.027	0.513		Open spaces = Low	−0.51	3.032	0.082	0.603
	No. of street crossing = Between 4 and 9	Reference Category					Open spaces = Medium	Reference Category			
	Accessibility to PT = High	−1.05	5.854	0.016	0.35		Intercept	3.971	2.991	0.084	
	Accessibility to PT = Low	−1.617	12.67	<0.001	0.198		Father’s commute mode choice = he doesn’t work	−1.235	2.848	0.091	0.291
	Accessibility to PT = Medium	Reference Category					Father’s commute mode choice = public transport	Reference Category			
	Population density = Low	1.132	4.406	0.036	3.1		Shopping in the neighborhood = 50−50	0.704	3.683	0.055	2.022
	Population density = Medium	Reference Category					Shopping in the neighborhood = never	1.755	4.735	0.03	5.783
	Open spaces = High	−0.941	3.143	0.076	0.39		Shopping in the neighborhood = usually	Reference Category			
	Open spaces = Low	−1.206	8.725	0.003	0.299		Entertainment in the neighborhood = never	−1.585	7.594	0.006	0.205
	Open spaces = Medium	Reference Category				siblings/close relatives/others	Entertainment in the neighborhood = usually	Reference Category			
	Intercept	5.9	9.732	0.002			Child’s travel to school mode = bike	−3.661	12.96	<0.001	0.026
mother	Age = 9	2.062	28.69	<0.001	7.863		Child’s travel to school mode = by foot	-1.939	11.09	0.001	0.144
	Age = 10	1.494	25.79	<0.001	4.453		Child’s travel to school mode = by public transport	−2.589	12.43	<0.001	0.075
	Age = 11	0.893	8.56	0.003	2.442		Child’s travel to school mode = own car	Reference Category			
	Age = 12	Reference Category					Parents’ perception of safety = moderate	−1.271	2.93	0.087	0.28
	Mother’s commute mode choice = by foot	−1.047	6.573	0.01	0.351		Parents’ perception of safety = unsafe	−2.019	8.041	0.005	0.133
	Mother’s commute mode choice = public transport	−1.157	10	0.002	0.314		Parents’ perception of safety = very unsafe	Reference Category			
	Mother’s commute mode choice = she doesn’t work	Reference Category					No. Children in the household = 1	−1.238	6.636	0.01	0.29
	Entertainment in the neighborhood = never	−1.199	10.99	0.001	0.301		No. Children in the household = 2−3	Reference Category			
	Entertainment in the neighborhood = sometimes	−0.906	12.46	<0.001	0.404		Income > =4001€	−1.667	3.921	0.048	0.189
	Entertainment in the neighborhood = usually	Reference Category					Income = 501−4000	Reference Category			
	Child’s travel to school mode = bike	−3.904	42.23	<0.001	0.02		Driving license = 1	0.572	4.495	0.034	1.771
	Child’s travel to school mode = by foot	−3.72	54.77	<0.001	0.024		Driving license = 2 or more	Reference Category			
	Child’s travel to school mode = by private/school service	−3.234	23.23	<0.001	0.039		No. of street crossing < =3	−0.769	7.54	0.006	0.463
	Child’s travel to school mode = by public transport	−5.044	66.59	<0.001	0.006		No. of street crossing = Between 4 and 9	Reference Category			
	Child’s travel to school mode = own car	Reference Category					Street connectivity = Low	0.771	2.854	0.091	2.162
	Child’s perception of safety = safe	−1.384	6.428	0.011	0.251		Street connectivity = Medium	Reference Category			
	Child’s perception of safety = very safe	−1.781	7.429	0.006	0.168		Population density = High	−1.989	11.44	0.001	0.137
	Child’s perception of safety = very unsafe	Reference Category					Population density = Medium	Reference Category			
	Child’s perception of security = insecure	−1.125	3.353	0.067	0.325						
